# Implementation and evaluation of family-based interventions within the Germany-wide Children of Mentally Ill Parents-network: study protocol for three prospective, rater-blinded, cluster-randomized controlled multicenter trials

**DOI:** 10.3389/fpsyt.2025.1735421

**Published:** 2026-03-19

**Authors:** Theresa Paumen, Anna Leidger, Joana Ralfs, Anna Modarressi, Ann-Kathrin Ozga, Annika Möhl, Anne Daubmann, Antonia Zapf, Tamara Waldmann, Carolin von Gottberg, Reinhold Kilian, Nicolas Pardey, Jan Zeidler, Timo Beeker, Sebastian von Peter, Martin Heinze, Silke Pawils, Anna K. Georg, Svenja Taubner, Olga Piwkowska, Sibylle M. Winter, Ruth Lingnau, Carina Stammann, Gerald Willms, Farhad Rezvani, Jörg Dirmaier, Koralia Sekler, Birgit Görres, Jürgen Gallinat, Martin Lambert, Sarah Hohmann, Martin Driessen, Michael Siniatchkin, Frank Jessen, Stephan Bender, Felix Bermpohl, Andreas Heinz, Christoph U. Correll, Christine Rummel-Kluge, Ulrich W. Seidl, Eva Möhler, Katharina Domschke, Christian Fleischhaker, Markus Jäger, Michele Noterdaeme, Frank Guderian, Tomasz Antoni Jarczok, Michael Kölch, Roland Burghardt, Klaus-Thomas Kronmüller, Fabian Klein, Martin Holtmann, Andreas J. Fallgatter, Tobias J. Renner, Gerd Schulte-Körne, Belinda Platt, Thomas Frodl, Hans-Henning Flechtner, Christian Haase, Stephanie Mehl, Katja Becker, Sylvia Claus, Tina In-Albon, Ralf Schlößer, Ekkehart Englert, Thomas Becker, Andreas Reif, Michael Berner, Simone Born, Meike Bottlender, Ursula Marschall, Luisa Schäfer, Sophia Rocabado, Thomas Krull, Michél Henkel, Alice Brinkmann, Jana Lübke, Heike Plachetka, Steve Hüttmann, Florian Brandt, Silke Wiegand-Grefe

**Affiliations:** 1 Department of Psychiatry and Psychotherapy, University Medical Center Hamburg-Eppendorf, Hamburg, Germany; 2 Institute of Medical Biometry and Epidemiology, University Medical Center Hamburg-Eppendorf, Hamburg, Germany; 3 University of Ulm, Department of Psychiatry II, Section: Health Economics and Health Services Research, Bezirkskrankenhaus Günzburg, Günzburg, Germany; 4 Center for Health Economics Research Hannover (CHERH), Leibniz University Hannover, Hanover, Germany; 5 Center for Mental Health, Immanuel Klinik Rüdersdorf, University Hospital of Brandenburg Medical School Theodor Fontane, Rüdersdorf, Germany; 6 Department of Medical Psychology, University Medical Center Hamburg-Eppendorf, Hamburg, Germany; 7 Institute for Psychosocial Prevention, Centre for Psychosocial Medicine, University Hospital Heidelberg, Heidelberg, Germany; 8 Clinical Psychology and Psychotherapy of Childhood and Adolescence, University of Tübingen, Tübingen, Germany; 9 German Center for Mental Health (DZPG), Berlin, Germany; 10 Charité – Universitätsmedizin Berlin, Department of Child and Adolescent Psychiatry, Psychotherapy and Psychosomatics, Berlin, Germany; 11 Department of Evaluation and Implementation Research, aQua Institute for Applied Quality Improvement and Research in Health Care GmbH, Göttingen, Germany; 12 AFET Federal Association for Educational Assistance, Registered Association, Hanover, Germany; 13 Community Psychiatry Umbrella Organization, Registered Association, Cologne, Germany; 14 Department of Child and Adolescent Psychiatry and Psychotherapy, University Medical Center Hamburg-Eppendorf, Hamburg, Germany; 15 Evangelisches Klinikum Bethel gGmbH, Department of Psychiatry and Psychotherapy, University Medical Center Ostwestfalen-Lippe (OWL), Bielefeld University, Bielefeld, Germany; 16 University Clinic for Child and Adolescent Psychiatry and Psychotherapy, Protestant Hospital Bethel, University Clinics Ostwestfalen-Lippe, Bielefeld, Germany; 17 Clinic of Child and Adolescent Psychiatry, Psychosomatics and Psychotherapy, University Hospital Aachen, Rheinisch-Westfälische Technische Hochschule (RWTH) Aachen University, Aachen, Germany; 18 Department of Psychiatry, Medical Faculty, University of Cologne, Cologne, Germany; 19 Faculty of Medicine and University Hospital Cologne, Department of Child and Adolescent Psychiatry, Psychosomatics and Psychotherapy, University of Cologne, Cologne, Germany; 20 Department of Psychiatry and Psychotherapy, Charité Campus St. Hedwig Hospital, Berlin, Germany; 21 Department of Psychiatry and Psychotherapy, Charité Universitätsmedizin Berlin, Berlin, Germany; 22 Department of Psychiatry and Psychotherapy, Medical Faculty, Leipzig University, Leipzig, Germany; 23 Department of Psychiatry and Psychotherapy, Saarland-Heilstätten GmbH (SHG)-Kliniken Saarbrücken, Saarbrücken, Germany; 24 Department of Child and Adolescent Psychiatry, Psychosomatics and Psychotherapy, Saarland University Medical Center, Homburg, Germany; 25 Department of Psychiatry and Psychotherapy, Medical Center – University of Freiburg, Faculty of Medicine, University of Freiburg, Freiburg, Germany; 26 Department of Child and Adolescent Psychiatry and Psychotherapy, University Medical Center Freiburg, Freiburg, Germany; 27 Department of Psychiatry, Psychotherapy and Psychosomatic, District Hospital Kempten, Kempten, Germany; 28 Department of Child and Adolescent Psychiatry and Psychotherapy KJF Klinik Josefinum, Augsburg, Germany; 29 Department of Child and Adolescent Psychiatry, Neurology, Psychosomatics and Psychotherapy, Rostock University Medical Center, Rostock, Germany; 30 Department of Child and Adolescent Psychiatry, Klinikum Frankfurt/Oder, Frankfurt, Germany; 31 Child and Adolescent Psychiatry, Oberberg Klinik Fasanenkiez, Berlin, Germany; 32 Department of General Psychiatry and Psychotherapy, Landschaftsverband Westfalen-Lippe (LWL) Hospital Gütersloh, Gütersloh, Germany; 33 LWL-University Hospital for Child and Adolescent Psychiatry, Ruhr-University Bochum, Hamm, Germany; 34 Department of Psychiatry and Psychotherapy, University Hospital of Tübingen, Tübingen, Germany; 35 German Center for Mental Health (DZPG), partner site Tübingen, Tübingen, Germany; 36 Department of Child and Adolescent Psychiatry, Psychosomatics and Psychotherapy, University Hospital of Psychiatry and Psychotherapy, Tübingen, Germany; 37 Department of Child and Adolescent Psychiatry, Psychosomatics and Psychotherapy, LMU University Hospital, LMU Munich, Munich, Germany; 38 Department of Psychiatry and Psychotherapy and Center for Behavioral Brain Sciences, Otto von Guericke University of Magdeburg, Magdeburg, Germany; 39 Clinic for Child and Adolescent Psychiatry and Psychotherapy, University of Magdeburg, Magdeburg, Germany; 40 Clinic for Child and Adolescent Psychiatry, Psychosomatics and Psychotherapy, Helios Clinics Schwerin, Schwerin, Germany; 41 Department of Psychiatry and Psychotherapy & Center for Mind, Brain and Behavior, University of Marburg (Philipps Universität), Marburg, Germany; 42 Department of Child and Adolescent Psychiatry, Psychosomatics and Psychotherapy, University Hospital of Marburg and University of Marburg (Philipps Universität), Marburg, Germany; 43 Department of Psychiatry, Psychosomatics und Psychotherapy, Pfalzklinikum, Klingenmünster, Germany; 44 Clinical Child and Adolescent Psychology and Psychotherapy, University of Kaiserslautern-Landau, Landau, Germany; 45 Clinical Child and Adolescent Psychology and Psychotherapy, University of Mannheim, Mannheim, Germany; 46 Clinic for Psychiatry, Psychotherapy and Psychosomatics, Helios Hospital Erfurt, Erfurt, Germany; 47 Clinic for Child and Adolescent Psychiatry, Psychotherapy and Psychosomatics, Helios Hospital Erfurt, Erfurt, Germany; 48 Department of Psychiatry II, Ulm University, Ulm, Germany; 49 Department of Psychiatry, Psychosomatic Medicine and Psychotherapy, Goethe University Frankfurt, Frankfurt am Main, Germany; 50 Psychiatry and Psychotherapeutic Medicine, Municipal Clinical Center of Karlsruhe, Karlsruhe, Germany; 51 Child and Adolescent Psychiatry, Psychosomatics and Psychotherapy, Municipal Clinical Center of Karlsruhe, Karlsruhe, Germany; 52 Statutory Health Insurance, BARMER, Wuppertal, Germany; 53 Statutory Health Insurance, Techniker Krankenkasse, Hamburg, Germany; 54 Statutory Health Insurance, Deutsche Angestellten-Krankenkasse(DAK)-Gesundheit, Hamburg, Germany; 55 Statutory Health Insurance, Mobil Krankenkasse, Celle, Germany; 56 Statutory Health Insurance, Kaufmännische Krankenkasse Halle (KKH) Kaufmännische Krankenkasse, Hanover, Germany; 57 Statutory Health Insurance, Allgemeine Ortskrankenkasse Baden-Württemberg, Stuttgart, Germany; 58 Statutory Health Insurance, Allgemeine Ortskrankenkasse (AOK) - Die Gesundheitskasse in Hessen, Bad Homburg, Germany; 59 Statutory Health Insurance, Innungskrankenkasse (IKK) classic, Dresden, Germany; 60 Statutory Health Insurance, Innungskrankenkasse (IKK) Südwest, Saarbrücken, Germany; 61 The German Center for Child and Adolescent Health (DZKJ), Hamburg, Germany

**Keywords:** family therapy, new forms of care, psychodynamic work with families, psychosocial prevention, psychotherapy research, transgenerational mental illness

## Abstract

**Introduction:**

Children of mentally ill parents have an increased risk of developing a mental illness. From an ethical and health-economic perspective, psychotherapeutic care for this risk group is necessary to counter the risk of transgenerational transmission of mental illness. The psychosocial situation of affected families is complex and requires customized support. Within the Children-of-Mentally-Ill-Parents-Network (CHIMPS-NET), three family-based, needs-tailored new forms of care (NFC) – based on the manualized CHIMPS intervention – are implemented and evaluated at 21 locations in Germany. The online intervention iCHIMPS is described in a separate study protocol.

**Methods:**

For each NFC, a prospective, rater-blinded, cluster-randomized, controlled study is conducted. Data is collected from the perspective of both parents, all children aged 8 years and older, external raters and therapists at four measurement points: at baseline (T1) and 6 (T2), 12 (T3) and 18 (T4) months after randomization. Allocation to the respective trials is based on baseline assessments of children’s mental health symptoms/diagnoses and family functionality, which is followed by randomization. We hypothesize that children in the intervention groups (IGs) have fewer parent-reported mental health problems at T3 than in the respective control groups (CGs), which receive Treatment-as-Usual (TAU). The biometric effect evaluation is supplemented by health economic evaluations and a qualitative evaluation.

**Discussion:**

CHIMPS-NET has both raised awareness for children of mentally ill parents and enabled various stakeholders to network with each other. The network contributes to evidence-based care for this risk group. An update of the CHIMPS manual regarding the customized NFC is in process.

**Clinical trial registration:**

https://www.bfarm.de/DE/Das-BfArM/Aufgaben/Deutsches-Register-Klinischer-Studien/_node.html; DRKS00020380; https://www.clinicaltrials.gov: NCT04369625.

## Introduction

Children of mentally ill parents (CHIMPS) have a greatly increased risk of developing a mental illness themselves (1–4). Perinatal depression and anxiety in mothers are adversely associated with children’s development (5), and growing up with a mentally ill parent is associated with adverse psychological, social and physical outcomes in adulthood (6, 7). Factors which particularly nuance the adversity of the parental mental illness have been identified. Parental strategies of coping correlate significantly with children’s mental health and health-related quality of life: The more pronounced depressive coping strategies are, the more internalizing problems the children have (8). Lower social support for the children is associated with a lower health-related quality of life (9) and more internalizing problems (10). Finally, family dysfunctionality is associated with increased internalizing (10, 11) and externalizing (11) problems in the children.

Although parental mental illness is associated with generally increased stress for all children, they are each affected to different degrees. While some children are stressed, but mentally healthy, well-adjusted and resilient, others have mental health problems bordering on pathology, and lastly, other children have mental health problems that are within the range of a diagnosis (11–13). On the parents’ side, the situation is similarly complex. There is often one parent who is viewed as primarily mentally ill, while the second parent is either healthy, or has mental health problems without a diagnosis and without insight into the illness, or has a diagnosis of mental illness themselves. Remarkably, the presence of two mentally ill parents increases the risk of lower mental health in children (14). Initially, the psychosocial situation of affected families is heterogeneous (15, 16). The challenge of providing care for the target group of children of mentally ill parents and their families is to consider their heterogeneous situation and to offer tailored help for each individual child and their parents (17).

However, a patient’s parenthood is often neglected in German routine clinical care due to the individual-centered approach of the healthcare system. Affected children often do not receive adequate preventive or therapeutic support (18, 19), although children’s increased risk of mental illness can be countered by preventive protective factors and interventions (20–22). Despite the increasing relevance of the topic and the development of interventions (23–26), there is still a lack of high-quality studies on the effectiveness of interventions for families in which children grow up with mentally ill parents (27, 28). The development and evaluation of effective interventions is necessary to counter the transgenerational transmission of mental illnesses (29, 30), and to transfer these interventions into routine clinical care financed by health insurance.

From both an ethical (1–7) and health-economic (31) perspective, it is advisable to screen children of mentally ill parents for mental health symptoms and to treat them – if necessary – according to their needs as part of routine clinical care. This screening should be carried out as early as possible, ideally when parents first approach mental health services, to prevent the chronicity of mental health symptoms and the development of mental illness (19).

The central goals of the CHIMPS-NET are the implementation, evaluation and transfer of three family-based, needs-tailored new forms of care (NFC) – CHIMPS-Therapy (CHIMPS-T), CHIMPS-Multifamily-Therapy (CHIMPS-MFT) and CHIMPS-Prevention (CHIMPS-P) – for children of mentally ill parents and their families in every federal state in Germany. If the interventions prove to be successful, they could be incorporated into routine clinical care throughout Germany.

For a health economic evaluation of the CHIMPS interventions, we conduct an incremental cost-utility analysis. We analyze whether an increase in quality-adjusted life years (QALYs) is achieved by implementing the respective intervention, compared to Treatment-as-Usual (TAU), and whether potential additional costs associated with an increase in QALYs do not exceed a hypothetical threshold value, the maximum willingness to pay.

The aim of a qualitative evaluation is to interview parents and children regarding the following key questions:

1. What subjectively relevant changes in their everyday life result for children and parents from participation in CHIMPS-NET?
2. How does CHIMPS-NET change family dynamics?
3. How does CHIMPS-NET influence the understanding of and dealing with one’s own mental illness or risk status?
4. How do participants experience participation in CHIMPS-NET (process experience) and what significance does this have for the outcome from a subjective perspective?

Additionally, there is an online intervention named iCHIMPS for adolescents with mentally ill parents which is coordinated and evaluated at a study center in Ulm and is described in detail in a separate study protocol (32).

The research focus is to evaluate whether the three NFC will effectively improve children’s mental health. Each intervention group (IG) is compared with a respective control group (CG) who receives TAU at three follow-up assessments. The following hypotheses for CHIMPS-T, CHIMPS-MFT and CHIMPS-P are put forward.

The children in the IG show a larger decrease in psychological symptoms compared to children in the respective CG, measured with the German version of the Child Behavior Checklist [CBCL/1½-5 (33, 34); CBCL/6-18R (35, 36)] as change of the raw total problem score from baseline to T3.

The following hypotheses are formulated prospectively for secondary endpoints.

All interventions: The children in the IG experience fewer psychological symptoms, measured with the German version of the CBCL/1½-5 (33, 34) and CBCL/6-18R (35, 36) as raw subscale problem scores, after the intervention than at baseline. Children in the IG improve more than children in the respective CG. The effects remain stable across follow-up assessments.

Only CHIMPS-T and CHIMPS-MFT study: Fewer children in the IG are diagnosed with a mental illness, assessed with the German version of the Schedule for Affective Disorders and Schizophrenia for School-Age Children-Present and Lifetime Version [K-SADS-PL (37, 38)], after the intervention than at baseline. The number of children diagnosed in the IG decreases more than in the CG. The effects remain stable across follow-up assessments.

Only CHIMPS-P study: The health-related quality of life, measured with the German version of the KIDSCREEN-27 (39), of children in the IG is higher after the intervention than at baseline. Children in the IG improve more than children in the CG. The effects remain stable across follow-up assessments.

## Methods

Due to difficulties in recruitment, mainly due to the Covid-19 pandemic, some amendments to the study protocol were necessary during the course of the study. The protocol is presented here in its current version (based on an evaluation concept dated October 16, 2023). For reasons of transparency, all adjustments to the original protocol are presented as **Supplementary Material**.

### Design

**Figure 1:**
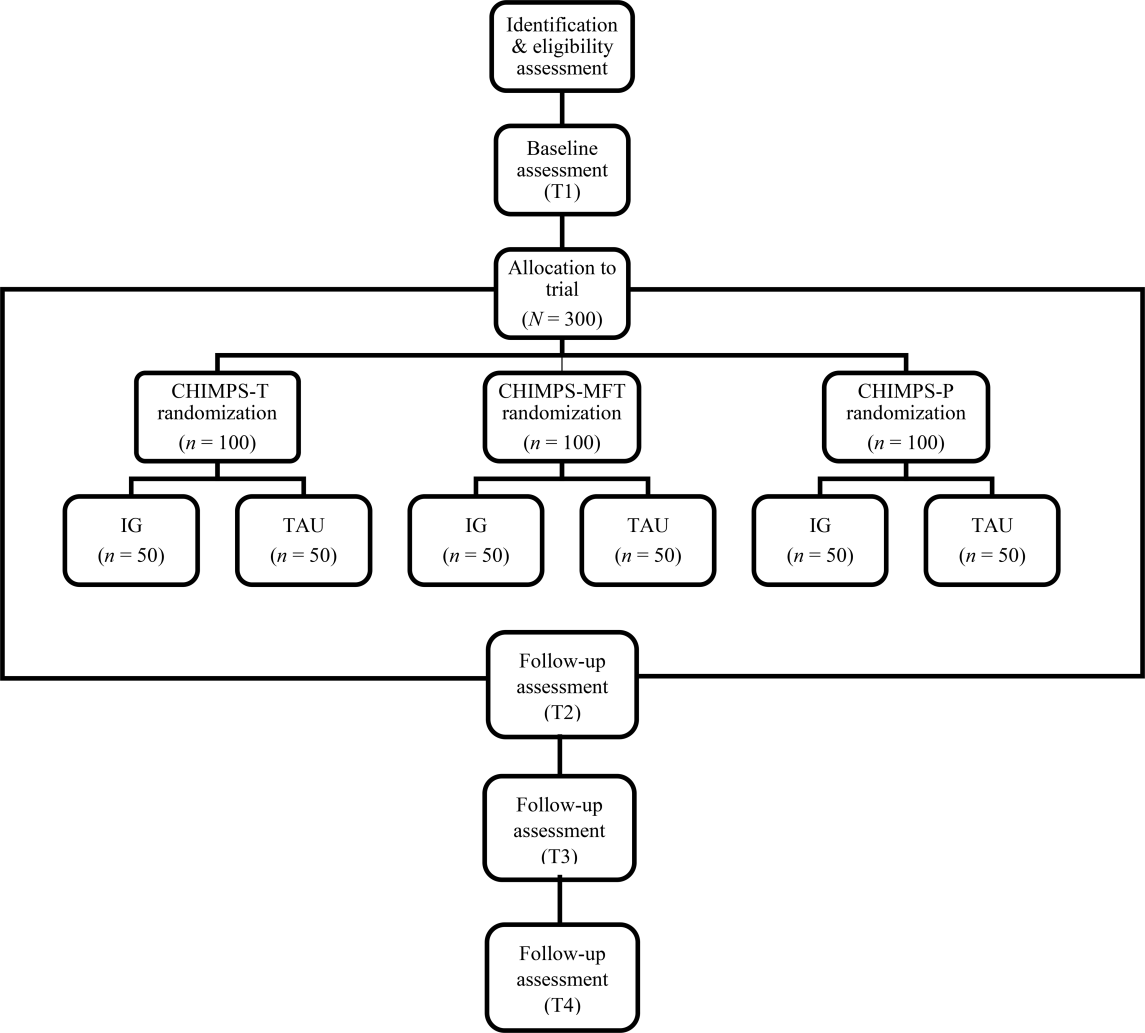
Study design of CHIMPS-NET.

The study is conducted over a period of 48 months (2020–2023). The study design includes a separate cluster-randomized controlled trial for each of the three interventions CHIMPS-T, CHIMPS-MFT and CHIMPS-P. Each intervention is tailored to a different target population and requires a respective CG that meets the respective eligibility criteria and is therefore comparable. The endpoints are assessed at four measurement points: before randomization (T1) and 6 (T2), 12 (T3) and 18 (T4) months after randomization. The intervention – if applicable – is scheduled to take place between T1 and T2. Data is collected from the perspective of the mentally ill parent, the other parent (if available), each child aged 8 years and older (self-assessment) and professionals (psychological diagnosticians and therapists). Both the IG and CG receive feedback on the results of the psychological diagnostics after each measurement point. If a child in the CG receives a diagnosis of mental illness, the family is informed about psychotherapeutic and psychiatric services available in routine clinical care. The design of the study is shown in **Figure 1**.

#### Consortium

The CHIMPS-NET consortium consists of the consortium leadership and consortium and cooperation partners from the following sectors: health insurance, public relations, community psychiatry and youth welfare, online-based interventions development, implementation research, evaluation, external data monitoring and data management and, throughout the project, 21 study centers in 13 federal states in Germany, both adult psychiatric clinics and child and adolescent psychiatric clinics. The consortium leadership is responsible for the central management of the network. To support the consortium leadership in decision-making processes, a steering committee and a steering group are formed in which all consortium and cooperation partners are represented.

#### Randomization procedure

The Institute of Medical Biometry and Epidemiology at the University Medical Center Hamburg-Eppendorf provides computerized randomization lists stratified by study center. Each randomized controlled trial (CHIMPS-T, CHIMPS-MFT, CHIMPS-P) is treated separately. The families form the sampling units and are randomized with an allocation ratio of 1:1 into the IG or into the CG with variable block length. The coordinating study center in Hamburg performs allocation based on the randomization lists. A family is randomized after completion of the baseline assessments and assignment to one of the three trials.

### Participants

#### Sample size and power calculation

The sample size and power calculations were performed with the sample size software PASS 15 (40). We used the modules *Tests for Two Means in a Cluster-Randomized Design* and *Tests for Two Proportions in a Cluster-Randomized Design*. The calculation of the sample size was based on the assumption that a family has an average of two children. We assumed that 25%/55%/15%/5% of families have one/two/three/four or more children. This resulted in a coefficient of variation for family size of 39%. In order to demonstrate a small to medium effect (Cohen’s *d* = 0.49) between the IG and the CG at T3, a total of 70 families (35 families per group), i.e., a total of 140 children (70 per group), are required. A power of 80%, a Type I error of 5% (two-sided hypothesis) and an intraclass correlation (ICC) coefficient of 5% were assumed. Assuming a drop-out rate of 30% of families, a total of 100 families (50 families per group), i.e., a total of 200 children (100 children per group), has to be recruited. The assumptions for calculating the sample size were the same for all three NFC and the calculated sample size applies to each randomized controlled trial. However, recruitment for CHIMPS-P is proving particularly difficult, and only a total of 34 families (17 families per group) are expected to be recruited. Anticipating a drop-out rate of 30% of families, data from a total of 22 families (11 families per group) will be available at the end of the study. With this sample, we can demonstrate an effect size (Cohen’s *d*) of 0.886.

#### Recruitment

Recruitment for the samples assigned to CHIMPS-T and CHIMPS-MFT will be completed by November 30, 2022, while recruitment for the sample assigned to CHIMPS-P will be finalized by March 31, 2023. Eligible families for all studies are typically identified via parents with mental illness who receive treatment at a psychiatry and psychotherapy clinic at the participating study centers. Additionally, families who learn about the study via flyers, the study website or other recommendations can contact the study team by e-mail. Of the 300 families targeted for recruitment, it is anticipated that about one third will show significantly low family functioning and will be assigned to the CHIMPS-T intervention vs. TAU. Another third is expected to demonstrate medium family functioning and thus be assigned to the CHIMPS-MFT intervention vs. TAU. For the final third, high family functioning and assignment to the CHIMPS-P intervention vs. TAU are expected.

#### Eligibility criteria

The target group is families with at least one parent with a mental illness (F1, F2, F3, F4, F5, F6 in the ICD-10) and with at least one child between the ages of 3 and 18 years. Further inclusion criteria for the three studies are regular contact between participating children and their mentally ill parent, consent to participate in the study and sufficient knowledge of the German language. Families of all health insurances are included. In addition, the families have to consent to data processing by the participating health insurances and to participation in so-called special care (the latter only applies to families assigned to an IG). The inclusion criteria for the three studies differ with regard to family functioning and the initial mental health symptoms of the children: The inclusion criterion for CHIMPS-T is an assessment of very low family functioning, measured using the Global Assessment of Relational Functioning scale [GARF scale (41); scores ≤ 20]. The inclusion criterion for CHIMPS-MFT is an assessment of medium family functioning [GARF scale (41) scores 21-80]. Families with high family functioning [GARF scale (41) scores > 80] are assigned to CHIMPS-MFT if at least one child meets a cut-off score in the CBCL (33–36) of ≥ 60 or a child receives a diagnosis according to the K-SADS-PL (37, 38). The inclusion criterion for CHIMPS-P is an assessment of high family functioning [GARF scale (41) scores > 80], as well as CBCL (33–36) scores below the cut-off value of 60 and no diagnosis according to the K-SADS-PL (37, 38). Exclusion criteria for all studies are mental illnesses of parents or children with severe acute symptoms, such as acute suicidality, active substance use, or acute psychotic symptoms that cannot be adequately treated with an outpatient intervention and instead indicate inpatient treatment.

### Interventions

The Children-of-Mentally-Ill-Parents-Network (CHIMPS-NET) is an interdisciplinary association in which three family-based, needs-tailored NFC were developed. All interventions are based on the manual *Kinder und ihre psychisch kranken Eltern: Familienorientierte Prävention – Der CHIMPs-Beratungsansatz* [Children and their mentally ill parents: Family-oriented prevention – The CHIMPs counseling approach (42)]. The manual’s clear, case-related description enables nationwide application and transfer of the interventions. All interventions are psychodynamic in nature. In line with this, the focus is on understanding the underlying causes of symptoms and problems. The most commonly used intervention techniques (clarifying, confronting and interpreting) originate from psychodynamic work. However, psychoeducational elements are integrated where necessary. CHIMPS-T is aimed at a group of highly dysfunctional, socially isolated families with children who often have a diagnosis of mental illness. CHIMPS-MFT is aimed at a group of moderately functional, hardly socially integrated families with children who often have psychological symptoms bordering on pathology. CHIMPS-P is aimed at a group of highly functional, socially integrated families with mostly well-adjusted, healthy children.

All interventions can easily be integrated into the families’ everyday lives and are implemented in cross-sectoral, interdisciplinary care structures.

#### CHIMPS-T

The CHIMPS-T intervention is a family-oriented, short-term psychoanalytic-based therapy for families with mentally ill parents. CHIMPS-T is applicable across all parental diagnoses of mental illness and is designed for a broad age range of children. For methodological reasons, however, the age range of children in this study is defined as 3–18 years. The intervention comprises an average of eight sessions per family (depending on the number of children) over six months: one initial therapy session of 90 minutes, two therapy sessions of 90 minutes with the parents, one therapy session of 50 minutes per child and three therapy sessions of 90 minutes with the whole family. The protected space of the individual sessions is intended to enable individuals to talk about issues that would not be addressed in a family setting. The initial sessions with parents aim to establish a psychotherapeutic relationship to address potential hesitations of introducing their children to the mental health care system. Sessions take place at low frequency every two to three weeks so that the appointments can be easily integrated into the families’ everyday lives. While the earlier CHIMPS approach (42) was initially developed as a preventive measure, CHIMPS-T is an intensified adaptation and is defined as a psychotherapeutic intervention due to the high symptom load of the children and adolescents. To this end, CHIMPS-T was combined with approaches from Transference-focused Psychotherapy [TFP (43)]. Topics of the therapeutic sessions include the family’s coping with the parental illness, family relationships (couple relationship, parent-child relationship, relationship with the family of origin, social network), as well as previous and future professional help. The focus is on the mental health and health-related quality of life of all family members in the context of parental mental illness.

#### CHIMPS-MFT

The CHIMPS-MFT intervention is a family-oriented, short-term psychoanalytic multi-family therapy for families with mentally ill parents. The target group regarding parental diagnoses and age range of the children is the same as for CHIMPS-T. The intervention comprises an average of eight sessions per family (depending on the number of children) over six months: one initial therapy session of 90 minutes per family and – if necessary – a further individual family session of 90 minutes, five family group therapy sessions of 120 minutes each with usually four (minimum three/maximum five) families and a final therapy session of 90 minutes per family. Sessions take place at a low frequency every two to three weeks so that the appointments can be easily integrated into the families’ everyday lives. The topics and focus of the therapeutic sessions are the same as for CHIMPS-T.

#### CHIMPS-P

The CHIMPS-P intervention is a family-oriented, short-term prevention for families with mentally ill parents. The target group in terms of parental diagnoses and age range of the children is the same as for CHIMPS-T and CHIMPS-MFT. The intervention comprises three sessions per family over two to three months: Families receive three preventive sessions of 90 minutes with the whole family. Sessions take place at low frequency every two to three weeks so that the appointments can be easily integrated into the families’ everyday lives. These three family sessions are based on the Finnish model *Let’s Talk About Children* (44) and the Norwegian model *Child Talks* (45). For CHIMPS-P, the Finnish model was adapted as *Let’s Talk****With****Children*. The topics and focus of the preventive sessions are the same as for CHIMPS-T and CHIMPS-MFT.

#### CG (TAU)

TAU means that families in the CGs are free to receive any treatment customary in routine clinical care. The participants provide information on other treatments as part of the psychological diagnostics.

### Endpoints

An overview of the endpoints collected, including the source from which they are collected and the time at which they are collected, can be seen in **Table 1**.

**Table 1:** Endpoints collected in CHIMPS-NET.

Construct	Assessment tool	Source					Measurement point			
		Mentally ill parent	Other parent	Child (≥ 8 years)	Psychological diagnostician	Therapist	T1	T2	T3	T4
Demographics	*Ad-hoc* items	x	x	x			x	x	x	x
Mental health (children)	CBCL/1½-5, CBCL/6-18R	x	x				x	x	x	x
	K-SADS-PL				x		x	x	x	x
	YSR/11-18R			x^a^			x	x	x	x
	GAF scale				x		x	x	x	x
Mental health (parents)	BSI	x	x				x	x	x	x
	GAD-7	x	x				x	x	x	x
	PHQ	x	x				x	x	x	x
	M.I.N.I.				x		x			
	GAF scale				x		x	x	x	x
Health-related quality of life (children)	KIDSCREEN-27	x	x	x^a^			x	x	x	x
	EQ-5D-Y-3L			x			x	x	x	x
	EQ-5D-Y-3L Proxy	x	x				x	x	x	x
	SSRMI-short			x^a^			x	x	x	x
Health-related quality of life (parents)	SF-12	x	x				x	x	x	x
	EQ-5D-3L	x	x				x	x	x	x
Coping	RSQ	x	x				x	x	x	x
	EFK	x					x	x	x	x
Social support	OSSS-3	x	x	x^a^			x	x	x	x
Family relationships	GARF scale				x		x	x	x	x
	FB-A	x	x	x^a^			x	x	x	x
	ESI			x^a^			x	x	x	x
Goals and treatment satisfaction	FBB^b^	x	x	x^a^		x		x	x	x
	ZUF-8^b^	x	x	x^a^				x	x	x
	NEQ^b^	x	x	x^a^				x	x	x
	*Ad-hoc* items	x	x	x^a^		x	x	x	x	x
Process of intervention	CPPS^b^					x		x		
	WAI-SR^b^					x		x		
Intervention costs	CAMHSRI				x		x	x	x	x
	CSSRI				x		x	x	x	x

BSI, Brief Symptom Inventory; CAMHSRI, Children and Adolescent Mental Health Service Receipt Inventory; CBCL, Child Behavior Checklist; CPPS, Comparative Psychotherapy Process Scale; CSSRI, Client Sociodemographic and Service Receipt Inventory; ESI, Erziehungsstil-Inventar (parenting style questionnaire); EFK, Essener Fragebogen zur Krankheitsverarbeitung (coping questionnaire); FB-A, Allgemeiner Familienbogen (family relationships questionnaire); FBB, Fragebögen zur Beurteilung der Behandlung (treatment assessment questionnaire); GAD-7, Generalized Anxiety Disorder Scale-7; GAF scale, Global Assessment of Functioning scale; GARF scale, Global Assessment of Relational Functioning scale; K-SADS-PL, Kiddie Schedule for Affective Disorders and Schizophrenia – Present and Lifetime Version; M.I.N.I., Mini-International Neuropsychiatric Interview; NEQ, Negative Effects Questionnaire; OSSS-3, Oslo 3 Social Support Scale; PHQ, Patient Health Questionnaire; RSQ, Responses to Stress Questionnaire; SF-12, Short Form-12; SSRMI-short, Self-Stigma in Relatives of people with Mental Illness scale-short; WAI-SR, Working Alliance Inventory-short revised; YSR, Youth Self-Report; ZUF-8, Fragebogen zur Patientenzufriedenheit (patient satisfaction questionnaire).

a Only assessed in children ≥ 10 years old.

b Only assessed in intervention groups.

#### Primary endpoint

The primary, clinically relevant endpoint of the three studies is the children’s psychological symptoms reported by parents as change from baseline to the second follow-up assessment (T3), which are determined using the raw total problem score of the CBCL/1½-5 (33, 34) and of the CBCL/6-18R (35, 36) respectively. The CBCL/1½-5 (33, 34) is a parent-rated questionnaire for children aged 1.5 to 5 years, the CBCL/6-18R (35, 36) is a parent-rated questionnaire for children aged 6 to 18 years. The CBCL total problem score is a continuous endpoint and is operationalized as a change from baseline assessment. The CBCL/1½-5 (33, 34) and CBCL/6-18R (35, 36) raw total problem scores will be standardized before they will be combined.

#### Secondary endpoints

The psychiatric diagnoses and mental health of the children are further assessed using the German version of the K-SADS-PL (37, 38), the German version of the Youth Self-Report 11-18R [YSR/11-18R (35, 36)] and the Global Assessment of Functioning scale [GAF scale (46)]. The parents’ subjective symptom impairment is assessed using the German version of the Brief Symptom Inventory [BSI (47, 48)], the German version of the Generalized Anxiety Disorder Scale-7 [GAD-7 (49, 50)] and the German version of the Patient Health Questionnaire [PHQ (51, 52)]. The parents’ psychiatric diagnoses and psychological symptoms are assessed using the German version of the Mini-International Neuropsychiatric Interview [M.I.N.I (53, 54).] and the GAF scale (46). The children’s health-related quality of life is assessed with the German version of the KIDSCREEN-27 (39), the German version of the EQ-5D-Y-3L (55) or the EQ-5D-Y-3L Proxy (55) and a German translation of the Self-Stigma in Relatives of people with Mental Illness scale-short [SSRMI-short (56)], that of the parents with the German version of the Short Form-12 [SF-12 (57, 58)] and the German version of the EQ-5D-3L (59, 60). Children’s coping is assessed using a German translation of the Responses to Stress Questionnaire [RSQ (61)], that of the parents using the German coping questionnaire Essener Fragebogen zur Krankheitsverarbeitung [EFK (62)]. Social support is assessed using a German translation of the Oslo 3 Social Support Scale [OSSS-3 (63, 64)]. Family relationships are assessed using the GARF scale (41), the German family relationships questionnaire Allgemeiner Familienbogen [FB-A (65)] and the German parenting style questionnaire Das Erziehungsstil-Inventar [ESI (66)]. Goals and treatment satisfaction are measured using the German treatment assessment questionnaires Fragebögen zur Beurteilung der Behandlung [FBB (67)], the German patient satisfaction questionnaire Fragebogen zur Patientenzufriedenheit [ZUF-8 (68)], the German version of the Negative Effects Questionnaire [NEQ (69)] and *ad-hoc* items. The process of the intervention is assessed using a German translation of the Comparative Psychotherapy Process Scale [CPPS (70)] and the German version of the Working Alliance Inventory-short revised [WAI-SR (71, 72)], both completed by therapists. Intervention costs of the children are measured with the German version of the Children and Adolescent Mental Health Service Receipt Inventory [CAMHSRI (73)], those of the parents with the German version of the Client Sociodemographic and Service Receipt Inventory [CSSRI (74, 75)]. *Ad-hoc* items are presented as **Supplementary Material**.

### Data assessment and data management

At study entry, all family members from whom data is collected complete informed consent forms. For the baseline assessment (T1), both the mentally ill parent and the second parent – if they take part in the assessment – fill out questionnaires and complete a diagnostic interview about their child or children.

All children aged 8 years and older fill out questionnaires. All children aged 10 years and over further take part in a diagnostic interview about themselves. As soon as all questionnaires and diagnostic interviews are completed, the family is assigned to either the CHIMPS-T, CHIMPS-MFT or CHIMPS-P trial and then randomly assigned to either the corresponding IG or CG. The research staff subsequently informs the family about their group assignment (CHIMPS-T IG, CHIMPS-T CG, CHIMPS-MFT IG, CHIMPS-MFT CG, CHIMPS-P IG or CHIMPS-P CG) and provides feedback on the results of the diagnostic interviews. If the family is allocated to one of the IGs, the study therapist is informed and the intervention is initiated promptly. The intervention is scheduled to be completed before the follow-up assessments. If the family is allocated to one of the CGs, no further treatment is provided within the study. Follow-up assessments are carried out 6 (T2), 12 (T3) and 18 (T4) months after randomization. The families are contacted by the research staff and again fill out questionnaires and complete diagnostic interviews. Adverse events are recorded in parallel with the diagnostic follow-up interviews. In this study, adverse events are defined as (a) suicidality, (b) death, (c) self-harm, (d) hospitalization/emergency admission, e) other burdens. The families are paid an expense allowance of 50€ for participating in the study. The allowance is paid at the end of the study to increase the families’ motivation to fully complete the study.

Study data (original questionnaires and scans) of each participating study center are collected and managed by the coordinating center in Hamburg after the end of data collection. Data is collected on paper and entered into the electronic case report form provided by secuTrial^®^, which is designed by the Clinical Trial Center North (CTC North). The electronic data system is password-protected and only accessible to the study staff. The data is entered independently by two researchers and the double data entry is then compared by a third researcher. In collaboration with CTC North, automated rules for detecting inconsistencies in the data are integrated and validated by a defined data validation plan. All study-related information is stored securely at the respective study centers. All records that contain names or other personal identifiers, such as informed consent forms, are stored separately from study records identified by code numbers. Furthermore, a data delivery of secondary health data (e.g., treatments, costs) is agreed on and carried out by the participating health insurances. Site visits to all participating study centers are conducted by the CTC North – and later by the Data Management and Trust Center GmbH (DMATC GmbH) – for quality control regarding study protocol adherence. Any change to the study protocol is recorded in a formal amendment and requires agreement by the German Aerospace Center Deutsches Zentrum für Luft- und Raumfahrt (DLR), as well as approval by the local Ethics Committee, Hamburg Medical Council, and the Ethics Committees of all participating study centers prior to implementation. The main study outcomes concerning the primary and secondary endpoints will be published in peer-reviewed journals and presented at scientific conferences. All papers and abstracts must be approved by the coordinating study center before submission. The data analysis is presented as **Supplementary Material**.

## Discussion

This study protocol depicts the attempt to implement three family-based, needs-tailored interventions for children of mentally ill parents and their families in routine clinical care across Germany. The interventions’ long-term effectiveness and cost-effectiveness are evaluated based on cluster-randomized controlled trials with assessments at four measurement points. We hypothesize that the CHIMPS interventions will improve children’s mental health and health-related quality of life compared to the respective CGs. Health economic evaluations will provide further important information for decision-makers in the healthcare system, and a qualitative evaluation will enable us to map particularly specific, individual changes generated by the interventions.

Due to the trans-diagnostic nature of the interventions, a broad spectrum of families will be included in the study. Moreover, not only biological parents but also adoptive parents, foster parents or step-parents will be included (76). A wide range of outcomes for both children and parents (mental health, health-related quality of life, coping, social support, family relationships, intervention costs, assessment of intervention, satisfaction and goals, process of the intervention) is recorded in a multi-perspective manner (77, 78), allowing for a particularly comprehensive analysis of the current situation of children of mentally ill parents in Germany. An important implication for practice is that all children and all parents (i.e., even parents not previously identified as mentally ill) participating in the study are screened for mental health problems at baseline, so that all family members can be referred to appropriate support services if necessary. The CHIMPS interventions involve all family members. This aspect of the intervention represents an important strength, as preventive interventions addressing parents and children jointly produce larger effects compared to interventions addressing parents and children individually (28).

Cluster effects due to the family and multicenter structure are taken into account statistically. Since allocation to one of the three interventions is based on family functioning and the mental health of the children, which families are informed about after the baseline assessment, it cannot be ruled out that this severity classification influences the family members’ ratings at subsequent measurement points. The different backgrounds of the therapists who carry out the CHIMPS intervention could cause problems in terms of manual adherence. To check manual adherence, the therapy sessions will be documented. The comparison with a CG receiving TAU accepts a certain variability in the control group (79). However, the forms of care received by the families assigned to TAU are documented in diagnostic interviews. In addition, the comparison with a CG that receives TAU allows a particularly strong test of the interventions’ effectiveness. However, a limitation is that no minimum level of TAU has been defined. Even if the mentally ill parents have already been in contact with the healthcare system, it is possible that no family member of families in a CG receives any form of care during the study period. In this case, the IG would not be compared with another form of care, but with no care at all. As the majority of the families are recruited via mentally ill parents who receive inpatient treatment, it must be expected that the sample consists of particularly stressed families who may have difficulties with adherence to therapy and/or the study. Therefore, a strong focus should be placed on transparent and empathic communication with the participants in order to increase adherence (80). Regular meetings of the research staff of all study centers are held to discuss recruitment challenges. A focus will be placed on being present in the participating adult psychiatric clinics during recruitment to raise awareness of the CHIMPS-NET program.

### Conclusion and relevance

The three prospective, rater-blinded, cluster-randomized, controlled multicenter trials of CHIMPS-NET will expand the range of psychosocial care services available to children of mentally ill parents, address factors involved in the transgenerational transmission of mental disorders, and facilitate the adaptation of existing support programs for families with parental mental health problems and diagnoses (81).

The results of this study will be used to revise the CHIMPS manual (42). The three customized NFC will be included and described in the manual. If the effectiveness of the interventions is demonstrated, consideration should be given to adapting the interventions for other samples, e.g., children with (severely) physically ill parents.

The aim of evaluating the interventions is to integrate them into German routine clinical care in the long term. Funding of the interventions in routine clinical care beyond the duration of the study will be discussed with representatives of the health insurances as part of the study. These interventions would be the first scientifically evaluated services in routine clinical care for the risk group of children of mentally ill parents (20). Care for this target group should be comprehensive and integrative and address the whole family in order to meet their complex psychosocial and health requirements (82, 83).

## Supplementary material

Supplementary Table 1 Amendments to the original study protocol.
