# Supplementary material for: Implementation and evaluation of family-based interventions within the Germany-wide Children of Mentally Ill Parents-network: study protocol for three prospective, rater-blinded, cluster-randomized controlled multicenter trials
Source: Front Psychiatry. 2026 Mar 19;16:1735421. doi: 10.3389/fpsyt.2025.1735421 (PMC13045562; doi:10.3389/fpsyt.2025.1735421)
Supplement: Supplementary Table 1 — Amendments to the original study protocol. [file DataSheet1.docx]

**Supplementary material 1.** Amendments to the original study protocol

|  | Original protocol content | Amendment |
| --- | --- | --- |
| Recruitment period and duration | 01.04.2020-30.03.2021  (12 months) | CHIMPS-T and CHIMPS-MFT: 01.06.2020-30.11.2022 (30 months);  CHIMPS-P:  01.06.2020-31.03.2023 (34 months) |
| Follow-up period | Fourth quarter of 2020 to fourth quarter of 2022 | Fourth quarter of 2020 to fourth quarter of 2023 |
| Measurement points | Before randomization (T1) and 6 (T2), 12 (T3) and 18 (T4) months after randomization | In order to carry out at least three assessments for all families despite an extended recruitment period, the follow-up period is shortened in some cases from 6 to 5 or 4 months, and the T4 measurement is omitted for families randomized from December 2022 onwards.  The following measurement points change:   - Randomization in June 2022 - T4 October 2023 - Randomization in July 2022 - T3 June 2023, T4 October 2023 - Randomization in August 2022 - T3 June 2023, T4 October 2023 - Randomization in September 2022 - T3 July 2023, T4 November 2023 - Randomization in December 2022 - T2 May 2023, T3 November 2023 - Randomization January 2023 - T2 June 2023, T3 October 2023 - Randomization February 2023 - T2 July 2023, T3 November 2023 - Randomization March 2023 - T2 July 2023, T3 November 2023 |
| Primary measurement point | T4 | T3 (due to the extended recruitment period, the T4 asessment can only be carried out in a subsample) |
| Questionnaires and diagnostic interviews | Ad-hoc items, BSI, CAMHSRI-DE, CBCL/1½-5, CBCL/6-18R, CSSRI-DE, EBI, EFK, ESI, EQ-5D-3L, EQ-5D-Y-3L, EQ-5D-Y-3L Proxy, FB-A, FBB, GAD-7, GAF scale, GARF scale, KIDSCREEN-27, K-SADS-PL, OSSS-3, PHQ, RSQ, SF-12, CPPS, WAI-SR, YSR/11-18R, ZUF-8 | Additionally M.I.N.I., NEQ, SSRMI-short.  EBI was eliminated. |
| Sample | The calculation of the sample size is based on the assumption that a family has an average of two children. We assume that 25%/55%/15%/5% of families have one/two/three/four or more children. This results in a coefficient of variation for family size of 39%. In order to demonstrate a small to medium effect (Cohen's *d* = 0.29) between the IG and the CG at T3, a total of 200 families (100 families per group), i.e., a total of 400 children (200 children per group), are required. A power of 80%, a type I error of 5% (two-sided hypothesis) and an intraclass correlation (ICC) coefficient of 5% are assumed. Anticipating a drop-out rate of 30% of families, a total of 286 families (143 families per group), i.e., a total of 572 children (286 children per group) has to be recruited. The assumptions for calculating the sample size are the same for all three trials, so a total of 858 families must be included in the calculations at T4. Based on clinical experience indicating that our sample consists of particularly burdened families, we anticipate that a further 30% of families drop out, so that initially 1200 families are included. | In order to demonstrate a small to medium effect (Cohen's *d* = 0.49) between the IG and the CG at T3, a total of 70 families (35 families per group), i.e., a total of 140 children (70 per group), are required. A power of 80%, a type I error of 5% (two-sided hypothesis) and an intraclass correlation (ICC) coefficient of 5% are assumed. Anticipating a drop-out rate of 30% of families, a total of 100 families (50 families per group), i.e., a total of 200 children (100 children per group), has to be recruited. The assumptions for calculating the sample size are the same for all three trials and the calculated sample size applies to each randomized controlled trial. However, recruitment for CHIMPS-P was proving particularly difficult, and only a total of 34 families (17 families per group) are expected to be recruited. Anticipating a drop-out rate of 30% of families, data from a total of 22 families (11 families per group) are available at the end of the study. With this sample, we can demonstrate an effect size (Cohen's *d*) of 0.886. |
| Legal foundation | Contract for so-called special care in accordance with § 140a Sozialgesetzbuch (SGB; German Social Code) V on the psychosocial care of children and adolescents (selective contract). | Extended by § 630a Bürgerliches Gesetzbuch (BGB; German Civil Code) to include families who are insured with health insurances not participating in the selective contract. |
| Intervention period | The last intervention is scheduled to start in the second quarter of 2021 and end in the fourth quarter of 2021. | The last intervention is scheduled to start in the fourth quarter of 2022 and end in the second quarter of 2023. |
| Format of interventions | All interventions are face-to-face interventions. | Covid-19 pandemic-related restrictions in face-to-face format. Depending on regional regulations and technical requirements, study centers choose the best possible alternative format for interventions, e.g., video conferencing. Documentation of the format used for each intervention unit (face-to-face, video, telephone).  We expect the observed effects to be independent of the format and test this assumption in a subgroup analysis. |

Abbreviations: BSI, Brief Symptom Inventory; CAMHSRI, Children and Adolescent Mental Health Service Receipt Inventory; CBCL, Child Behavior Checklist; CPPS, Comparative Psychotherapy Process Scale; CSSRI, Client Sociodemographic and Service Receipt Inventory; EBI , Eltern-Belastungs-Inventar (parent stress inventory); ESI, Erziehungsstil-Inventar (parenting style questionnaire); EFK, Essener Fragebogen zur Krankheitsverarbeitung (coping questionnaire); FB-A, Allgemeiner Familienbogen (family relationships questionnaire); FBB, Fragebögen zur Beurteilung der Behandlung (treatment assessment questionnaire); GAD-7, Generalized Anxiety Disorder Scale-7; GAF scale, Global Assessment of Functioning scale; GARF scale, Global Assessment of Relational Functioning scale; K-SADS-PL, Kiddie Schedule for Affective Disorders and Schizophrenia – Present and Lifetime Version; M.I.N.I., Mini-International Neuropsychiatric Interview; NEQ, Negative Effects Questionnaire; OSSS-3, Oslo 3 Social Support Scale; PHQ, Patient Health Questionnaire; RSQ, Responses to Stress Questionnaire; SF-12, Short Form-12; SSRMI-short, Self-Stigma in Relatives of people with Mental Illness scale-short; WAI-SR, Working Alliance Inventory-short revised; YSR, Youth Self-Report; ZUF-8, Fragebogen zur Patientenzufriedenheit (patient satisfaction questionnaire).
