# Supplementary material for: Implementation and evaluation of family-based interventions within the Germany-wide Children of Mentally Ill Parents-network: study protocol for three prospective, rater-blinded, cluster-randomized controlled multicenter trials
Source: Front Psychiatry. 2026 Mar 19;16:1735421. doi: 10.3389/fpsyt.2025.1735421 (PMC13045562; doi:10.3389/fpsyt.2025.1735421)
Supplement: Supplementary file 2 [file DataSheet2.docx]

**Supplementary material 2.** Ad-hoc items in German original as used in the study and their English translation

|  | Parent | Child (≥ 8 years)^a^ | Therapist |
| --- | --- | --- | --- |
| Demographics | Heutiges Datum  **Today’s date**  Ihr Geburtsdatum  **Your Date of birth**  Wer beantwortet den Fragebogen?   1. Erkrankter Elternteil 2. Partner/in   **Who answers the questionnaire?**   1. **Mentally ill parent** 2. **Partner**   Ihr Geschlecht   1. Weiblich 2. Männlich 3. Divers   **Your gender**   1. **Female** 2. **Male** 3. **Non-binary**   Wie viele Kinder haben Sie insgesamt?  **How many children do you have in total?**  Wie viele davon sind zwischen 0 und einschließlich 21 Jahren?  **How many of them are between the ages of 0 and 21 years?**  Ihr Familienstand   1. Ledig 2. Verheiratet 3. Mit Lebenspartner/in 4. Geschieden 5. Verwitwet   **Your marital status**   1. **Single** 2. **Married** 3. **In partnership** 4. **Divorced** 5. **Widowed**   Ihr höchster Schulabschluss   1. Hauptschule 2. Realschule 3. Abitur/Fachhochschulreife 4. Ohne Abschluss 5. Sonstiger Abschluss…   **Your highest school-leaving certificate**   1. **Main school** 2. **Middle school** 3. **A-levels/university entrance qualification** 4. **Without degree** 5. **Other degree**   In den letzten 12 Monaten waren/haben Sie   1. Vollzeit gearbeitet 2. Teilzeit gearbeitet 3. In Elternzeit 4. Student/Studentin 5. Krank/arbeitsunfähig/beurlaubt 6. Arbeitssuchend/arbeitslos   **In the last 12 months you have been/have**   1. **Worked full-time** 2. **Worked part-time** 3. **On parental leave** 4. **Student** 5. **Sick/unable to work/on leave due to…** 6. **Job seeker/unemployed**   Arbeiten Sie so viel Sie möchten?   1. Ja 2. Nein, ich würde gerne mehr arbeiten 3. Nein, ich würde gerne weniger arbeiten   **Do you work as much as you like?**   1. **Yes** 2. **No, I would like to work more** 3. **No, I would like to work less**   Leiden Sie derzeit an einer körperlichen Erkrankung?   1. Ich bin gesund 2. Ich leide an…   **Are you currently suffering from a physical illness?**   1. **I am healthy** 2. **I suffer from…**   Leiden Sie unter einer psychischen Erkrankung?   1. Ich bin derzeit gesund 2. Ich leide derzeit unter… seit… 3. Ich litt früher unter… von… bis…   **Do you suffer from a mental illness?**   1. **I am currently healthy** 2. **I am currently suffering from... since...** 3. **I used to suffer from... from... to...**   Sind Sie derzeit wegen psychischer Beschwerden in ambulanter Behandlung?   1. Nein 2. Ja, seit…   **Are you currently receiving outpatient treatment for mental health problems?**   1. **No** 2. **Yes, since…**   Wenn ja, wie oft sind Sie aktuell in ambulanter Behandlung?   1. Mehrmals pro Woche 2. 1 Mal pro Woche 3. 14-täglich 4. 1 Mal im Monat oder seltener   **If yes, how often are you currently receiving outpatient treatment?**   1. **Several times a week** 2. **Once a week** 3. **14-daily** 4. **Once a month or less**   Waren Sie schon einmal wegen psychischer Beschwerden in stationärer Behandlung?   1. Nein 2. Ja   Wenn ja, wie oft?  Wann war das letzte Mal?  **Have you ever received inpatient treatment for mental health problems?**   1. **No** 2. **Yes**   **If so, how often?**  **When was the last time?** | Heutiges Datum  **Today’s date**  Dein Geburtsdatum  **Your Date of birth**  Dein Geschlecht   1. Weiblich 2. Männlich 3. Divers   **Your gender**   1. **Female** 2. **Male** 3. **Non-binary**   In den letzten 12 Monaten war ich   1. Schüler/in 2. Auszubildende/r / Student/in 3. Krank 4. Angestellte/r 5. Arbeitssuchend/-los   **In the last 12 months I have been**   1. **Pupil** 2. **Apprentice / student** 3. **Sick** 4. **Employee** 5. **Jobseeker/unemployed**   Bist du ausreichend beschäftigt?   1. Ja 2. Nein, ich würde gerne mehr zur Schule gehen/studieren/arbeiten 3. Nein, ich würde gerne weniger zur Schule gehen/studieren/arbeiten   **Are you busy enough?**   1. **Yes** 2. **No, I would like to go to school/study/work more** 3. **No, I would like to go to school/study/work less**   Hast du psychische Probleme?   1. Nein 2. Ja   Wenn ja, welche?  **Do you have mental health problems?**   1. **No** 2. **Yes**   **If yes, which?**  Bist Du derzeit in ambulanter psychotherapeutischer Behandlung?   1. Ja, seit… 2. Nein   **Are you currently undergoing outpatient psychotherapeutic treatment?**   1. **Yes, since…** 2. **No**   Wenn ja, wie oft bist Du aktuell in ambulanter Behandlung?   1. Mehrmals pro Woche 2. 1 Mal pro Woche 3. 14-täglich 4. 1 Mal im Monat oder seltener   **If yes, how often are you currently receiving outpatient treatment?**   1. **Several times a week** 2. **Once a week** 3. **14-daily** 4. **Once a month or less** |  |
| Goals and treatment satisfaction | Bitte beschreiben Sie möglichst genau, was für Sie das wichtigste Ziel der Familienberatung war  **Please describe as precisely as possible what the most important goal of the family counseling was for you**  Haben Sie Ihr wichtigstes Beratungsziel der jetzigen Familienberatung erreicht?   1. Gar nicht erreicht 2. Sehr wenig erreicht 3. Wenig erreicht 4. Mittel erreicht 5. Hoch erreicht 6. Sehr hoch erreicht   **Have you achieved the most important goal of your current family counseling?**   1. **Not achieved at all** 2. **Very little achieved** 3. **Little achieved** 4. **Medium achieved** 5. **Highly achieved** 6. **Very highly achieved**   Haben Ihnen die Familiengespräche insgesamt geholfen?   1. Gar nicht 2. Sehr wenig 3. Wenig 4. Mittel 5. Hoch 6. Sehr hoch   **Did the family talks help you overall?**   1. **Not at all** 2. **Very little** 3. **Little** 4. **Medium** 5. **Highly** 6. **Very highly** | Bitte beschreibe möglichst genau, was für Dich das wichtigste Ziel der Familienberatung war  **Please describe as precisely as possible what the most important goal of the family counseling was for you**  Hast Du Dein wichtigstes Beratungsziel der jetzigen Familienberatung erreicht?   1. Gar nicht erreicht 2. Sehr wenig erreicht 3. Wenig erreicht 4. Mittel erreicht 5. Hoch erreicht 6. Sehr hoch erreicht   **Have you achieved the most important goal of your current family counseling?**   1. **Not achieved at all** 2. **Very little achieved** 3. **Little achieved** 4. **Medium achieved** 5. **Highly achieved** 6. **Very highly achieved**   Haben Dir die Familiengespräche insgesamt geholfen?   1. Gar nicht 2. Sehr wenig 3. Wenig 4. Mittel 5. Hoch 6. Sehr hoch   **Did the family talks help you overall?**   1. **Not at all** 2. **Very little** 3. **Little** 4. **Medium** 5. **Highly** 6. **Very highly** | Bitte beschreiben Sie möglichst genau, was für Sie das wichtigste Ziel der Familienberatung war  **Please describe as precisely as possible what the most important goal of the family counseling was for you**  Hat die Familie das wichtigste Beratungsziel erreicht?   1. Gar nicht erreicht 2. Sehr wenig erreicht 3. Wenig erreicht 4. Mittel erreicht 5. Hoch erreicht 6. Sehr hoch erreicht   **Has the family achieved the most important counseling goal?**   1. **Not achieved at all** 2. **Very little achieved** 3. **Little achieved** 4. **Medium achieved** 5. **Highly achieved** 6. **Very highly achieved**   Folgende Beratungssitzungen fanden statt:  Elterngespräche…  Kindergespräche…  Diagnostik…  Familiengespräche…  Gesamte Anzahl…  **The following counseling sessions took place:**  **Parent counseling...**  **Children counseling...**  **Diagnostics...**  **Family counselings...**  **Total number...**  Dauer der Behandlung:  Datum des Erstkontaktes…  Datum des letzten Kontaktes…  **Duration of treatment:**  **Date of first contact...**  **Date of last contact...**  Die Familie   1. hat die Beratung abgeschlossen. 2. hat die Beratung vorzeitig beendet/abgebrochen.   Grund für den Abbruch:  **The family**   1. **has completed the counseling.** 2. **has ended/cancelled the counseling prematurely.**   **Reason for termination:** |

^a^Ad-hoc items regarding goals and treatment satisfaction are only assessed in children ≥ 10 years old.
