# Supplementary material for: Implementation and evaluation of family-based interventions within the Germany-wide Children of Mentally Ill Parents-network: study protocol for three prospective, rater-blinded, cluster-randomized controlled multicenter trials
Source: Front Psychiatry. 2026 Mar 19;16:1735421. doi: 10.3389/fpsyt.2025.1735421 (PMC13045562; doi:10.3389/fpsyt.2025.1735421)
Supplement: Supplementary file 3 [file DataSheet3.docx]

**Supplementary material 3.** Data analysis

**Primary data analysis**

The family members’ baseline data are presented for each randomized controlled trial overall and for both groups (intervention and control). Continuous variables are summarized using the mean, standard deviation, median, minimum and maximum. Categorical variables are summarized using absolute and relative frequencies. In addition, the number of missing observations is given. No tests to check for statistically significant differences between groups at baseline are performed, but the clinical significance of any imbalance is noted. Furthermore, all psychiatric and somatic diagnoses according to ICD-10 (as well as the number of diagnoses) at the start of the study for parents and children are reported. The primary analysis is based on the Full Analysis Set (FAS). It is as complete as possible and as close to the Intention-to-Treat (ITT) principle as possible. The FAS includes all randomized families, regardless of whether they refuse the intervention or whether other study protocol violations occur. The Per Protocol (PP) population is a subpopulation of the FAS and includes families without major protocol violations.

Minor protocol deviations are defined as follows:

• Completion of the intervention after T2

• Advancing T3 and/or T4 by up to two months

• Mentally ill parent does not want to or cannot take part in the study

• Implementation of the intervention as an online intervention

• Change of therapists in the course of the intervention

Major protocol deviations are defined as follows:

• Accidental inclusion of ineligible families or family members

• Intervention or other procedure deviates from the protocol

• Intervention or other procedure deviates from randomization

• MFT with fewer than three families

• Families or family members receive only part of the intervention (in the case of CHIMPS-T and CHIMPS-MFT maximum 2 sessions; in the case of CHIMPS-P maximum 1 session)

• The intervention starts after T3

A linear mixed model is used for the primary analysis. The recruitment center, the family and the children are treated as nested random effects. For the CHIMPS-MFT trial, the MFT group is an additional random term, and an additional center is defined for families that come from different centers but are in the same MFT group (online format). Parents (mentally ill vs. second parent) are treated as fixed effects. A first-order autoregressive variance-covariance matrix of the residual matrix (measurement points) is considered. If it cannot be estimated, a compound symmetry variance-covariance matrix of the residual matrix (measurement points) is assumed. Treatment group, CBCL (33, 34, 35, 36) total raw score at baseline (T1), time, and the interaction between treatment group and time are included as fixed effects in the model. The primary outcome is operationalized as change from baseline. If the *p*-value of the interaction between treatment group and time is >0.15 (two-tailed hypothesis), the interaction is eliminated from the model.

The primary analysis is the group comparison between the IG and the CG for the CBCL (33, 34, 35, 36) total raw score at the 12-month follow-up (T3; change from baseline). Only this comparison, tested at a significance level of 5% (two-sided hypothesis), is considered confirmatory. This analysis for the primary endpoint is performed for all three randomized controlled trials. Because the three trials are independent samples, the Type I error does not need to be corrected for multiple testing.

All secondary outcomes are analyzed exploratively. Continuous secondary endpoints are analyzed in the same manner as the primary endpoint. Instead of the CBCL (33, 34, 35, 36) total raw score at baseline, the respective baseline score is included as a covariate in the model. For endpoints without a baseline score, the absolute scores at all measurement points are used and no adjustment for a baseline value is made. The fixed effect for parents is not included in the model when children provide information about themselves, or when external raters are asked about the children. When the parents provide information about themselves, two models (mentally ill parent vs. second parent) are built. Binary secondary outcomes are analyzed using a mixed logistic regression model when possible.

As sensitivity analyses, the primary endpoint analysis is repeated in the corresponding PP population.

Safety endpoints are analyzed using frequency tables based on the population of all randomized families.

Subgroup analyses are carried out: face-to-face vs. online format, urban vs. small town/rural study center, and low vs. medium vs. high socioeconomic status (index including weighted values for education, occupation and income). For this purpose, the primary analysis is repeated with an additional interaction term between the treatment group and the corresponding subgroup. The relevant *p*-value for concluding that there is an interaction between treatment group and subgroup is the *p*-value of the interaction. However, if the *p*-value of the interaction between treatment group and time in the primary analysis is <0.15, the three-way interaction between treatment group, time and subgroup is included.

For a post-hoc analysis of the primary endpoint, the CHIMPS-T IG and the CHIMPS-MFT IG are combined into one group and compared with the corresponding combined CG.

**Health economic evaluation of primary data**

We conduct an incremental cost-utility analysis from the perspective of the German health and social system based on the net-benefit approach. In addition to services provided by the health and social system assessed with the CAMHSRI (73), the intervention costs per child are calculated for each intervention. Service utilization is recorded retrospectively for six months at each of the four measurement points.

The incremental cost-effectiveness ratio (ICER) is calculated from the difference in total costs between IG and CG in relation to the difference in QALYs over the period of the study. The primary outcome of the health economic evaluation is the ICER. The calculation of QALYs is based on the EQ-5D-Y-3L (55).

If the baseline data show no selection bias with regard to total costs and QALYs, the cost difference and QALY difference are determined using linear regression models with robust standard errors. If the baseline data provide indications of a selection bias, the cost difference and QALY difference are determined using a Seemingly Unrelated Regressions (SUR) model with a corresponding adjustment for the baseline assessments. If there are no indications of a selection bias, the non-parametric bootstrapping method with 10,000 replications is used to estimate the stochastic uncertainty of the unadjusted cost-utility ratio. In both cases, the position of the unadjusted cost-utility ratio in the cost-effectiveness plane is determined first. Subsequently, the cost-effectiveness acceptability curve is used to determine the acceptance rate for a range of willingness to pay from 0€ to 125,000€.

Missing values are considered under the MAR assumption using a Full-Information-Maximum-Likelihood method (84). Imputation of values is carried out using the Last Observation Carried Forward (LOCF) method.

Absolute and relative frequencies are provided to describe categorical data. To describe continuous variables, mean, standard deviation, median, the 25% percentile, the 75% percentile, minimum and maximum are calculated.

**Health economic evaluation of secondary data**

We further conduct an incremental cost-utility analysis of secondary cost data (including costs for the outpatient sector, inpatient sector, institutional outpatient services, pharmaceutical care, remedies, rehabilitation, days of incapacity for work, sick pay) provided by the health insurances. Data from participants who were insured with one of the participating health insurances between January 1, 2019 and June 30, 2023 is evaluated. Intervention costs are calculated for each intervention. For the evaluation of costs, time periods are formed based on the dates of measurement points. If a measurement point is within a treatment period, costs are calculated on a daily basis for each period between two measurement points.

The primary outcome is the ICER, as in the health economic evaluation of primary data. QALYs are assessed using the EQ-5D-3L (58, 59) and the EQ-5D-Y-3L (55). If more than one person answers the questionnaire on a child, the arithmetic mean of the EQ-5D-Y-3L (55) score is used for descriptive presentation and further analysis. The costs of an intervention at family level are allocated to each family member individually, depending on the frequency of individual and joint sessions.

SUR models and non-parametric bootstrapping are used as in the health economic evaluation of primary data, and cost-effectiveness planes as well as cost-effectiveness acceptability curves are used to present and interpret the results.

As in the health economic evaluation of primary data, missing values are dealt with using the LOCF method.

Absolute and relative frequencies are provided to describe categorical data. To describe continuous variables, mean, standard deviation, median, the 25% percentile, the 75% percentile, minimum and maximum are calculated. In addition, the 10% winsorized group averages are given for total costs to minimize the impact of outliers and extreme values. Analysis of continuous variables is based on the Mann-Whitney U test. Data preparation and analysis, including cost-effectiveness planes and cost-effectiveness acceptability curves, are performed using SAS 9.4 for Windows (85). The estimation of SUR models and resulting constant ellipses are implemented using STATA (86).

The total ITT population includes all study participants for whom health insurance data is submitted.

The ITT population 2 includes all study participants for whom health insurance data is submitted and further requires EQ-5D-(Y-)3L (55, 59, 60) scores of at least two measurement points. In addition, secondary data provided by the health insurances is extracted including the year before randomization to check for cost differences between the respective IG and CG.

The Complete Case Analysis (CCA) population includes study participants for whom complete data sets of EQ-5D-(Y-)3L (55, 59, 60) scores are available for all four measurement points and secondary data provided by the health insurances is available for all measurement periods. In addition, secondary data provided by the health insurances is extracted including the year before randomization to check for cost differences between the respective IG and CG.

**Qualitative evaluation**

Four different interview situations are carried out:

• Group interviews with the entire family

• Individual interviews with the mentally ill parent

• Individual interviews with the second parent

• Individual interviews with children

Interview guidelines are adapted to the interview situation and intervention arm. Before the actual interview, general information (e.g., age, level of education, occupation, treatment experience) is collected. Interviews are carried out online via Webex (Cisco). Qualitative data collection takes place over a period of 24 months, from August 2021 to August 2023. The study centers are asked to check families for a basic willingness to participate in interviews after the end of the intervention and, if necessary, to forward contact details to the qualitative evaluation team. The aim is to conduct all interviews three to six months after full completion of the intervention so that intervention effects or the absence of effects will be clear enough and at the same time participation will be sufficiently well remembered. Interviews generally last around 60 minutes, although family interviews are often longer and individual interviews with children are significantly shorter.

The interview material is analyzed using two different qualitative methods, namely an analysis according to the Grounded Theory Methodology (GTM) and the Most Significant Change (MSC) approach. The GTM serves to develop or discover a theory from data in a rule-guided, controlled and verifiable manner. The theory developed should be firmly anchored (grounded) in empirical data. The core of the MSC approach is the writing of a change story, which depicts the changes personally experienced and subjectively assessed as most significant from the narrative perspective of a participating person.

# **References**

84. *Stata Statistical* Software. Version 17. StataCorp; 2021. https://www.stata.com/

85. *SAS/STAT Software*. Version 15.3. SAS Institute Inc.; 2023. https://documentation.sas.com/doc/en/pgmsascdc/9.4_3.5/statug/titlepage.htm

86. *Stata Statistical Software*. Version 16.1. StataCorp; 2020. https://www.stata.com/
